# Modeling Neurological Disorders with Human Pluripotent Stem Cell-Derived Astrocytes

**DOI:** 10.3390/ijms20163862

**Published:** 2019-08-08

**Authors:** Mika Suga, Takayuki Kondo, Haruhisa Inoue

**Affiliations:** 1 iPSC-based Drug Discovery and Development Team, RIKEN BioResource Research Center (BRC), Kyoto 619-0237, Japan; 2 Center for iPS Cell Research and Application (CiRA), Kyoto University, Kyoto 606-8507, Japan; 3 Medical-risk Avoidance based on iPS Cells Team, RIKEN Center for Advanced Intelligence Project (AIP), Kyoto 606-8507, Japan

**Keywords:** astrocytes, disease modeling, iPSC, induced pluripotent stem cells, glia, human neurological disorders

## Abstract

Astrocytes play vital roles in neurological disorders. The use of human induced pluripotent stem cell (iPSC)-derived astrocytes provides a chance to explore the contributions of astrocytes in human diseases. Here we review human iPSC-based models for neurological disorders associated with human astrocytes and discuss the points of each model.

## 1. Introduction

Astrocytes are the most abundant glial cell type in the central nerve system (CNS) and have multiple roles on neuronal development and function. Astrocytes regulate synaptogenesis, modulate synaptic plasticity, provide metabolic support to neurons, secrete or absorb neurotransmitters from synapses, regulate extracellular ion concentrations, support the brain–blood barrier (BBB), and promote myelination in the white matter. Conversely, their dysfunction has been implicated in several neurological disorders such as neurodegenerative diseases, neurodevelopmental diseases, epilepsy and astroglioma [1,2,3].

## 2. Diversity of Astrocyte Functions and Phenotypes

Astrocytes are extremely specialized and heterogeneous in terms of morphology and function throughout the CNS. According to classical taxonomy, astrocytes are divided into two major classes, protoplasmic astrocytes in the gray matter and fibrous astrocytes in the white matter [4]. Protoplasmic astrocytes have highly branched and complex processes that contact blood vessels and neurons, whereas fibrous astrocytes have elongated, more simple processes that contact blood vessels, oligodendrocytes, and axons at the nodes of Ranvier [5]. Astrocytes are coupled by gap junctions and form large networks [6]. Astrocytes also make contact and form gap-junction coupling with oligodendrocytes in different brain regions, termed panglial networks [7,8]. Recent studies found that astrocyte morphology and function diversify with age and location in the brain [2,9,10,11,12,13,14]. For example, mouse neocortical protoplasmic astrocytes within layer II–IV exhibit distinct morphologies, structural interactions with synapses, and molecular expressions [15]. How astrocytes acquire these different phenotypes during brain development and maturation is largely unknown.

Astrocytes acquire different reactive phenotypes when they respond to different pathological stimuli, such as infection, ischemia, neurodegenerative disorders, and aging. Reactive astrocytes have modulated gene expression profiles that regulate both structural and biochemical changes [9,11,16]. Two major different types of reactive astrocytes, A1 and A2, were shown to be induced by injury and disease in the adult CNS [17]. A1 astrocytes, which are induced by injury, neuroinflammation, and neurodegenerative disease, produce proinflammatory molecules and secrete molecules that are toxic to neural cells. Several cytokines including IL-1α, tumor necrosis factor (TNF) and C1q released by activated microglia can promote the formation of reactive A1 astrocytes such that the A1 astrocytes no longer promote neuronal survival, outgrowth, or synapse formation, nor phagocytize synapses or debris [6]. On the other hand, A2 astrocytes, which are induced by ischemia, secrete neuroprotective cytokines including thrombospondins (TSPs) to provide neurotrophic support and modulate inflammatory responses that promote neuronal survival and tissue repair [17]. Non-reactive astrocytes become reactive astrocytes with a spectrum of potential molecular, cellular, and functional changes, including neurotoxic or neuroprotective properties, in response to a wide range of extracellular signals and stress. Reactive changes in astrocyte properties occur in a context-dependent manner, and under each circumstance that would induce a continuum heterogeneous population of reactive astrocytes [18,19]. It is hypothesized that reactive astrocytes play a role not only in aging and age-related diseases including neurodegenerative disease, but also in neurodevelopmental diseases [9,16]. A1 astrocytes have been identified in the post-mortem brain tissues of individuals with neurological diseases, including Alzheimer’s disease (AD), Parkinson’s disease (PD), multiple sclerosis (MS), amyotrophic lateral sclerosis (ALS), and Huntington’s disease (HD). Astrocytes display different phenotypes depending on the pathogenesis [3]. Moreover, astrocyte reactivity is induced by normal aging [9]. It was shown that astrocytes in aged mouse brains express genes associated with reactivity, particularly in the hippocampus and striatum, which are areas linked to neurodegenerative diseases [13,16]. Thus, astrocyte phenotypic changes have been implicated in the pathogenesis of a wide range of neurological disorders.

## 3. Human PSC-Derived Astrocytes as In Vitro Models

Human and rodent astrocytes show significant differences [5,11,20], with human cortical astrocytes being larger, more complex and diverse [20]. For example, human cortical protoplasmic astrocytes are nearly 2.6-fold larger in diameter and extend 10-fold more glial fibrillary acidic protein (GFAP)-expressing primary processes than their rodent counterparts [20]. Furthermore, human cortical protoplasmic astrocytes propagate Ca^2+^ waves approximately 4-fold faster than rodent cortical protoplasmic astrocytes [20]. The two types also show distinct gene expression profiles [11]. These differences must be considered when studying animal models. However, there is limited access to astrocytes from patient brain samples. Human astrocytoma cell lines [21] and primary human adult and fetal astrocytes [11] are available as in vitro cellular models, but fail to fully recapitulate human diseases.

Human pluripotent stem cells (PSCs), which include human embryonic stem cells (ESCs) [22] and human induced pluripotent stem cells (iPSCs) [23], can be used to model neurological diseases. Several protocols exist for the directed differentiation of human PSCs toward astrocytes. Furthermore, human ESCs and iPSCs with gene modification mimicking the mutation of the inherited disease can be generated, as too can iPSCs from the patient without gene modification. Accordingly, several studies have used human PSCs to model astrocyte function or dysfunction in neurological diseases (Table 1).

## 4. Methods for Generating Human PSC-Derived Astrocytes

Since the derivation of neural precursors from human ESCs was reported in 2001 [41,42], various differentiation protocols for neural cells including astrocytes from human PSCs have been described [24,29,43,44,45,46,47,48,49,50]. Most of them were developed by recapturing embryonic developmental events, although astrocyte development is not fully understood. Protocols for generating astrocytes from human PSCs generally consist of four main steps: (1) Conversion of the undifferentiated human PSCs into rosette-forming neuroepithelial cells; (2) Induction of the neural stem cells with or without morphogens for regional patterning; (3) Glia lineage specification by long-term expansion of the induced neural stem cells either in adhesion or suspension culture with growth factors; and (4) Astrocyte terminal differentiation and maturation [43,51].

To obtain neural stem cells, the serum-free culture of embryoid body (SFEB) method [47] and the SFEB with quick aggregation (SFEBq) method [48] have been used as well as the dual inhibition of SMAD signaling in adherent human PSC cultures [52]. Alternatively, long-term self-renewing human iPSC-derived neuroepithelial stem cells (ltNES) [53] can be used for astrocyte differentiation as well as neuronal differentiation [49].

The combination of morphogens and growth factors, such as FGF8, RA, or SHH [54,55,56], were used for rostral-caudal and dorsal-ventral patterning during the induction and regional specification of neural stem cells [57]. The induced cells expressed distinct homeodomain transcription factors and displayed phenotypic differences [57]. Using these techniques, researchers separately generated region-specific astrocyte subtypes including spinal cord astrocytes, midbrain astrocytes, and neocortical astrocytes [43,57,58,59].

Astrocyte lineage specification and terminal differentiation are conducted with growth factor-rich undefined medium such as fetal bovine serum-containing medium, growth factor-defined medium or growth factor-reduced medium. The efficiency of the terminal differentiation is independent of the growth factors used. On the other hand, serum accelerates terminal differentiation. Although astrocytes can be expanded easily in vitro, they tend to undergo senescence with the exhaustion of growth factors [60,61]. Serum components also may change the astrocyte phenotype. Indeed, acutely purified primary astrocytes exhibit extensive process-bearing morphologies under serum-free media, but exhibit polygonal fibroblast-like morphologies under serum-containing media [11]. Furthermore, a serum-containing medium induces a phenotypic change and reactivity to human iPSC-derived astrocytes [35]. Thus, in vitro culture conditions should be controlled very carefully. Two groups [43,57] have described methods to generate functional astrocytes from human PSCs under serum-free conditions. The maturation of astrocytes is also important to recapitulate the physiological function or disease phenotype. Long-term cultivation as a three-dimensional (3D) structure can accelerate the maturation of iPSC-derived astrocytes. Sloan et al. conducted up to 20 months cultivation of iPSC-derived astrocytes in 3D cortical neurospheres. Astrocytes after long-time culture closely resembled primary human fetal astrocytes and showed similar global gene-expression patterns and functions to that of primary human astrocytes [62]. It is likely that the astrocyte function requires integration of the cells into a system. Yet, it is unknown if astrocytes in the entire brain form just a single (syncytium) or multiple networks (syncytia) through gap junctional coupling [63]. iPSC-derived astrocytes partially recapitulate functional networks by simply assessing calcium influx spread [43,49]. However, these systems lack a robust astrocytic network which can modulate neuronal activity, signal transmission, or vessel contraction. Additionally, it is the future target to define the “healthy or normal” character in Ca^2+^ oscillation or spread for disease modeling, because of the wide-variety of Ca^2+^ spreading mechanisms [64]. Therefore, it is the incoming step to construct a culture system, consisting of multiple cell types to mimic human brain and model disorders in the future.

Alternatively, the direct conversion of skin fibroblasts to astrocytes was reported [44]. The defined transcription factors nuclear factor I A (NFIA), nuclear factor I B (NFIB), and SRY-box transcription factor 9 (SOX9) induce an astrocytic phenotype in human fibroblasts [44]. In addition, a recent study demonstrated that human PSCs acquire glial fate by the transient forced activation of NFIA and SOX9 tandemly or NFIA alone, and further differentiated into functional astrocytes [46,65]. Understanding the molecular mechanisms in astrocyte specification will lead to improved molecular activation to enable PSC-derived astrocytes with better operability and better yield in the future.

## 5. Modeling Neurological Disorders Using Human PSC-Derived Astrocytes

Below we highlighted the modeling of several neurological disorders with human PSC-derived astrocytes.

### 5.1. Alzheimer’s Disease

Alzheimer’s disease (AD) is the most common dementia worldwide. In AD, astrocyte pathology is expressed even before neuronal death. Astrocytes in rodent AD models and AD patients become reactive, have aberrant calcium signaling, and change their gene expression profile and metabolism [3]. AD astrocytes display elevated levels of GFAP expression and GABA production and release [66]. Moreover, amyloid-β(Aβ) accumulates in AD astrocytes. Most cases of AD are late-onset sporadic AD, 1–2% are early-onset familial AD with underlying gene mutations in amyloid precursor protein (APP) or presenilin-1 and -2 (PSEN1/2) [67].

We generated iPSCs from one sporadic AD patient and two familial AD patients with APP E693Δ mutation and APP V717L mutation. The AD iPSCs were differentiated into neurons and astrocytes, in which Aβ oligomers were found to have accumulated [24]. We further used the AD iPSC model for drug screening and discovered that docosahexaenoic acid (DHA) may be effective for a subset of AD patients to reduce or prevent the symptoms [24].

Oksanen et al. generated iPSCs from three AD patients with PSEN1 exon 9 deletion and analyzed the AD iPSC-derived astrocytes [25]. The AD astrocytes exhibited increased Aβ production, altered cytokine release, and dysregulated Ca^2+^ homeostasis [25]. Furthermore, due to the altered metabolism, the AD astrocytes showed increased oxidative stress and reduced lactate secretion, as well as compromised neuronal supportive function, as evidenced by altering Ca^2+^ transients in the healthy neurons.

### 5.2. Parkinson’s Disease

Parkinson’s disease (PD) is the second most common neurodegenerative disease and is characterized by a significant loss of ventral midbrain dopaminergic neurons in the substantia nigra pars compacta. A pathological hallmark of PD is the accumulation of Lewy bodies (LBs), which mainly consist of α-synuclein, and α-synuclein aggregates spread from neuron to neuron in PD lesions [68]. Most PD cases are sporadic, but monogenic mutations in 17 genes have been identified and implicated in the PD pathogenesis [69]. Mutations in the leucine-rich repeat kinase 2 (LRRK2) gene are the most common cause of inherited PD, particularly the G2019S mutation in LRRK2 [69].

Di Domenico et al. generated iPSC-derived astrocytes and dopaminergic neurons from familial PD patients carrying the G2019S mutation and compared the cells with iPSC-derived astrocytes and dopaminergic neurons from healthy controls [26]. They demonstrated that α-synuclein, secreted from the PD iPSC-derived astrocytes, exerted neurotoxic function on surrounding dopaminergic neurons, leading to a non-cell-autonomous neuronal dysfunction. In addition, they identified dysfunctional chaperone-mediated autophagy (CMA), impaired macroautophagy, and progressive α-synuclein accumulation in the PD iPSC-derived astrocytes. Furthermore, the reactivation of CMA protected the PD iPSC-derived astrocytes and dopaminergic neurons via the clearance of α-synuclein accumulation [26]. These findings described a non-cell-autonomous contribution of astrocytes during PD pathogenesis.

### 5.3. Huntington’s Disease

Huntington’s disease (HD) is a progressive and fatal neurodegenerative disorder caused by the trinucleotide repeat expansion (CAG) in exon 1 of the huntingtin (HTT) gene. The CAG repeat expansion causes an expanded polyglutamine tract at the amino terminus of the HTT protein. The mutant HTT protein accumulates and aggregates not only in neurons, including medium spiny neurons of the striatum, but also in other neural cell types such as astrocytes in the brain of HD patients [70].

Juopperi et al. generated iPSCs from a father with adult-onset HD and 50 CAG repeats and his daughter with juvenile HD and 109 CAG repeats [27]. The astrocytes derived from the HD-specific iPSCs exhibited a large number of cytoplasmic, electron clear vacuoles a phenomenon seen in HD patients. The severity of this phenotype was dependent on the number of CAG repeats.

### 5.4. Tauopathy

Mutations in the gene encoding the microtubule-associated protein TAU (MAPT) are a common cause of frontotemporal dementia (FTD), a group of neurodegenerative diseases characterized by progressive nerve cell loss in the frontal lobes and the temporal lobes in the brain.

Hallmann et al. generated iPSCs from one FTD patient carrying the N279K MAPT mutation to establish a cell model of the astrocyte pathology in FTD. In their study, FTD iPSC-derived neurons and astrocytes were analyzed. The FTD astrocytes showed disease-associated changes in the TAU expression, increased vulnerability to oxidative stress and increased protein ubiquitination. Co-culture of healthy control neurons with FTD iPSC-derived astrocytes demonstrated that the astrocytes induced alterations in stress-response and gene-expression profiles in the neurons [28].

### 5.5. TDP-43 Proteinopathy

Transactive response DNA-binding protein (TDP-43) forms ubiquitinated inclusions in the cytoplasm and nuclei of neurons and astrocytes in patients with amyotrophic lateral sclerosis (ALS) or frontotemporal lobar dementia (FTLD). Consistently, mutations in the TDP-43 gene are a common cause of familial ALS and FTLD.

Serio et al. generated functional astrocytes from human iPSCs carrying a TDP-43 mutation and showed that the mutant TDP43 astrocytes exhibited increased levels of TDP-43, impaired subcellular localization of TDP-43, and decreased cell survival [29]. However, the mutant TDP-43 astrocytes did not adversely affect the survival of cocultured neurons, suggesting a glial cell-autonomous pathological phenotype is associated with the TDP-43 proteinopathy.

### 5.6. Amyotrophic Lateral Sclerosis

ALS is a group of rare neurological diseases characterized by the progressive degeneration of motor neurons, resulting in progressive muscle weakness and respiratory failure. More than 90% of ALS cases are considered sporadic, with the remaining considered familial. Among familial ALS, 20% are linked to various point mutations in the Cu/Zn superoxide dismutase 1 (SOD1) gene.

Wada et al. generated human ESCs overexpressing SOD1 mutant (G93A) by gene editing and then differentiated them to spinal motor neurons and astrocytes [30]. The astrocytes were found to secrete factors toxic to the spinal motor neurons [30]. Tyzack et al. showed that ephrin type-B receptor 1 (EphB1) is upregulated in injured motor neurons and that EphB1 induces astrocytic STAT3 signaling followed by a protective and anti-inflammatory signature in astrocytes [31]. They generated astrocytes from ALS patient-iPSCs carrying SOD1 mutation (D90A) and demonstrated that EphB1 and the downstream pathway is disrupted [31]. These ALS studies identified the increase of astrocytic toxicity and dysfunction of the neuroprotective astrocytic response.

### 5.7. Spinal Muscular Atrophy

Spinal muscular atrophy (SMA) is a group of childhood neurodegenerative disorders caused by loss or mutations in the survival motor neuron 1 (SMN1). SMA is characterized by a loss of motor neurons or anterior horn cells, resulting in progressive muscle weakness and muscle wasting in proximal muscles, respiratory difficulty, paralysis, and death.

McGivern et al. established iPSC-derived astrocytes from patients with SMA, and reported that the astrocytes had larger cell bodies and shorter processes, reduced GDNF expression, increased basal calcium levels and decreased response to ATP stimulation [32]. The SMA iPSC-derived astrocyte models suggested that reactive astrocytes are involved in motor neuron loss in SMA.

### 5.8. Alexander Disease

Alexander disease (AxD) is a rare inherited neurodegenerative disorder that primarily affects astrocytes. A variety of *GFAP* gene mutations causes astrocyte dysfunction in AxD [71,72]. The common neuropathological features of AxD are the degeneration of white matter (leukodystrophy) and the formation of cytoplasmic inclusions, termed Rosenthal fibers, within the astrocytes [71,72,73].

We generated iPSCs from three AxD patients and differentiated them into astrocytes, in which Rosenthal fiber-like structures and GFAP-positive aggregates were observed [33]. The AxD astrocytes produced and released more inflammatory cytokines than healthy control iPSC-derived astrocytes, suggesting that the AxD astrocyte models recapitulate the leukodystrophy and a variety of other AxD features [33]. Li et al. also established AxD astrocyte models from three AxD patient-iPSCs [34]. These iPS-derived astrocytes exhibited Rosenthal fiber-like structures and GFAP aggregation. Using a co-culture system of AxD iPS-derived astrocytes and oligodendrocytes, Li et al. found that the astrocytes secreted more molecules that inhibited oligodendrocyte progenitor cell function and impair myelination [34].

### 5.9. Multiple Sclerosis

Multiple sclerosis (MS) is an auto-inflammatory disease of the CNS. It is characterized by demyelination followed by axonal loss and neurodegeneration [74]. In MS, reactive astrocytes are present around the lesions [74,75], however, the pathogenesis and cause of MS is largely unknown.

Perriot et al. generated iPSCs from four MS patients [35]. They found IL-1b, TNF-a, and IL-6, all cytokines important for the neuroinflammation seen in MS, in a serum-free culture system triggered a specific reactivity in the iPSC-derived astrocytes.

### 5.10. Autism Spectrum Disorder

Autism spectrum disorder (ASD) is a group of neurodevelopmental disorders. A small percentage of ASD is syndromic ASD with copy number variants (CNVs) or mutations in ASD-risk genes, but most are non-syndromic ASD with unknown genetic etiology [76].

Russo et al. generated iPSCs from a clinically well-characterized cohort of three individuals with non-syndromic ASD sharing common behaviors [36]. Analyzing the synaptogenesis and neuronal activity of a mixed neuron culture derived from ASD iPSCs, they found that the neurons had a significant decrease in synaptic gene expression and protein levels, glutamate neurotransmitter release, and, consequently, reduced spontaneous firing rate. Moreover, co-culture experiments of the ASD iPSC-derived astrocytes and neurons revealed that cytokines including interleukin-6 (IL-6) secreted from the astrocytes, interfered with proper neuronal development.

### 5.11. Rett Syndrome

Rett syndrome (RTT) is a rare neurodevelopmental disorder caused by a variety of mutations in the Methyl-CpG-binding protein2 (*MECP2*) gene on the X chromosome. RTT patients show abnormalities in cognitive, sensory, emotional, motor and autonomic functions, but the disease mechanism is not well understood. Mouse models have shown that MeCP2 deficiency in astrocytes causes abnormal BDNF regulation, cytokine production, and neuronal dendritic induction [1,77].

Williams et al. generated iPSC lines from RTT patients carrying the V247X (valine 247 to stop codon, nonsense), R294X (arginine 294 to stop codon, nonsense) or R306C (arginine 306 to cysteine, missense) mutations [78] and differentiated them into astrocytes [37].

Neurons co-cultured with astrocytes derived from RTT iPSCs showed shorter neurite length and fewer terminal ends than if co-cultured with control astrocytes [37]. These phenotypes are consistent with RTT pathologies. Moreover, the study demonstrated that both insulin-like growth factor 1 (IGF1) and GPE (a peptide containing the first three amino acids of IGF-1) partially rescued the neuronal deficits caused by the RTT astrocytes. Andoh-Noda et al. generated two iPSC lines from RTT twins with a de novo frame-shift mutation in MECP2 (G269AfsX288) [38,79]. They demonstrated that the RTT iPSC lines did not express detectable MeCP2 protein during any stage of differentiation into neural cells and that MeCP2 deficiency triggers a change in the astrocytic gene expression, yielding accelerated astrocyte formation from RTT iPSC-derived neural stem cells [38]. Finally, Delépine et al. identified altered microtubule dynamics and impaired vesicular transport in RTT iPSC-derived astrocytes carrying the MECP2 gene mutation (R294X) [39]. These studies have shed light on astrocytic abnormalities in RTT and suggested that astrocytes might be a target of RTT therapy.

### 5.12. Epilepsy

Epilepsy is one of the common neurological disorder in which hypersynchronous neuronal firing occurs as a consequence of intensive burst activity from groups of neurons. Epilepsy can be genetic or acquired, although the cause is unknown in most cases. Childhood epileptic encephalopathies (CEEs) such as Dravet syndrome, Angelman syndrome, and Rett syndrome (RTT) are increasingly linked to specific genetic mutations. iPSC-derived neural cells, including GABAergic or glutamatergic neurons, have come to be a common tool to model epileptic disorders [80]. Astrocyte regulates neurotransmission by encasing thousands of synapses to form tripartite synapses between pre- and postsynaptic structures of two neurons and the surrounding astrocytic process [81]. Astrocytes also contact blood vessels and other glial cells, affecting neurotransmission. Therefore, astrocytes have a key role in the seizure activity, in addition to the well-established neurogenic mechanisms [3,82].

A total of 70–80% of patients with RTT develop seizures. As mentioned in earlier sections, astrocytes derived from iPSC of RTT patients do not have enough ability to support neurite outgrowth or synapse formation [37]. In the case of drug-induced epilepsy, single-cell transcriptome analysis of cocultured iPSC-derived neurons and astrocytes clarified that the iPSC-derived astrocyte upregulate the activities of AMPA and NMDA receptors, which is directly related to epileptiform discharge by gabazine or kaliotoxin [40].

## 6. Closing Remarks

Astrocytes play a key role in many neurological disorders, but details are still lacking. Astrocytes derived from human PSCs with disease-specific gene mutations can be a powerful tool to understanding the cellular phenotypes and disease mechanisms in vitro. These models are expected to clarify how astrocytes contribute to neurological disorders including the underlying mechanisms.

## Figures and Tables

**Table 1:** Modeling astrocyte contributions in neurological disorders with human pluripotent stem cells.

Disease	Source	Gene Mutation/Modification	Reference
Alzheimer’s disease (AD)	Patient derived iPSCs	APP E693Δ mutation APP V717L mutation Sporadic AD	Kondo et al., 2013 [24]
Alzheimer’s disease (AD)	Patient derived iPSCs	PSEN1 exon 9 deletion	Oksanen et al., 2017 [25]
Parkinson’s disease (PD)	Patient derived iPSCs	LRRK2 G2019S mutation	di Domenico et al., 2019 [26]
Huntington’s disease (HD)	Patient derived iPSCs	HTT CAG repeat expansion	Juopperi et al. 2012 [27]
Tauopathy	Patient derived iPSCs	MAPT N279K mutation	Hallmann et al. 2017 [28]
TDP-43 proteinopathy	Patient derived iPSCs	TDP-43 M337V mutation	Serio et al., 2013 [29]
Amyotrophic lateral sclerosis (ALS)	Human ESCs	SOD1 G93A overexpressing	Wada et al., 2012 [30]
Amyotrophic lateral sclerosis (ALS)	Patient derived iPSCs	SOD1 D90A mutation	Tyzack et al., 2017 [31]
Spinal muscular atrophy (SMA)	Patient derived iPSCs	SMN1 exons 7 and 8 deletion	McGivern et al., 2013 [32]
Alexander’s disease (AxD)	Patient derived iPSCs	GFAP R239C mutation GFAP E69K mutation GFAP L264P mutation	Kondo et al., 2016 [33]
Alexander’s disease (AxD)	Patient derived iPSCs	GFAP R239C mutation GFAP R79C mutation GFAP M73K mutation	Li et al., 2018 [34]
Multiple sclerosis (MS)	Patient derived iPSCs		Perriot et al., 2018 [35]
Autism spectrum disorder (ASD)	Patient derived iPSCs		Russo et al., 2018 [36]
Rett syndrome (RTT)	Patient derived iPSCs	MECP2 V247X mutation MECP2 R294X mutation MECP2 R306C mutation	Williams et al., 2014 [37]
Rett syndrome (RTT)	Patient derived iPSCs	MECP2 G269AfsX288 mutation	Andoh-Noda et al., 2015 [38]
Rett syndrome (RTT)	Patient derived iPSCs	MECP2 R294X mutation	Delépine et al., 2016 [39]
Drug-induced epilepsy	Human iPSCs		Ishii et al., 2017 [40]
